# The Influence of Milk Standardization on Chemical Composition, Fat and Protein Recovery, Yield and Sensory Properties of Croatian PGI Lički Škripavac Cheese

**DOI:** 10.3390/foods10040690

**Published:** 2021-03-24

**Authors:** Samir Kalit, Milna Tudor Kalit, Iva Dolenčić Špehar, Krešimir Salajpal, Dubravka Samaržija, Jasna Anušić, Ante Rako

**Affiliations:** 1 Department of Dairy Science, Faculty of Agriculture, University of Zagreb, Svetošimunska 25, 10000 Zagreb, Croatia; skalit@agr.hr (S.K.); ispehar@agr.hr (I.D.Š.); samarzija@agr.hr (D.S.); mlijeko@agr.hr (J.A.); 2 Department of Animal Science and Technology, Faculty of Agriculture, University of Zagreb, Svetošimunska 25, 10000 Zagreb, Croatia; ksalajpal@agr.hr; 3 Institute for Adriatic Crops and Karst Reclamation, Put Duilova 11, 21000 Split, Croatia; Ante.Rako@krs.hr

**Keywords:** milk standardization, skimmed milk powder, fat separation, fat and protein recovery, cheese yield, Lički škripavac cheese, soft cheese, sensory properties

## Abstract

The aim of this work was to evaluate the influence of cheese milk standardization on chemical composition, fat and protein recovery, yield and sensory properties of Croatian soft Protected Geographical Indication (PGI) Lički škripavac cheese. Standardization of milk to the casein/fat ratio of 0.7 was carried out by adding skimmed milk powder (SMP) to cheese milk and by skimming part of the milk fat. Results showed that losses of fat by whey were significantly (*p* < 0.05) lower after Lički škripavac cheese produced from standardized milk by skimming part of the milk fat. Standardization of cheese milk by addition of SMP caused higher losses of protein (*p* < 0.05) and total solids (*p* < 0.0001) by whey. Both methods of cheese milk standardization caused a significant (*p* < 0.01) decrease in milk fat and fat in dry matter content in cheese. In contrast, standardization of cheese milk caused a significant (*p* < 0.01) increase in protein content in cheese milk. Moisture in non-fat substance (MNFS) significantly (*p* < 0.05) decreased. Optimization of the casein/fat ratio did not cause a significant increase in fat recovery, but protein recovery significantly increased (*p* < 0.01). Addition of SMP to cheese milk significantly (*p* < 0.01) increased actual and adjusted cheese yield. The addition of SMP led to a noticeably higher (*p* = 0.10) sensory score of Lički škripavac cheese.

## 1. Introduction

The composition of cheese milk is a critical segment of cheese production influencing cheese yield and manufacturing efficiency (expressed as a percentage recovery of milk fat or protein to cheese), which are the main determinants of production success and profitability [1,2]. Milk fat is a source of components that are partially responsible for the flavor and aroma, as well as body of cheese, and noticeably responsible for cheese yield [3]. However, excessive amount of fat in cheese milk can negatively influence the composition and attributes of cheese curd due to the low whey excretion during the treatment of cheese curd or cheese grain [4]. On the contrary, low fat content in the cheese milk could cause firm, elastic and doughy texture of cheese made from such cheese milk [5].

As milk fat is the most variable milk component, due to many different reasons, such as season, stage of lactation, somatic cell count and feeding regime, this can lead to inconsistencies in cheese quality [1]. Industrial production of traditional cheeses is mainly based on traditional production processes, which are improved and adjusted to consumer’s requirements and modern production conditions with the aim of increasing production cost-effectiveness. Therefore, industrial cheesemaking includes standardization of milk, which means lowering the content of milk fat in relation to casein. According to that, the ratio proposed by Scott [6] of 0.7 was taken as a value for lowering the fat content in relation to the casein content in the production of Lički škripavac cheese, Croatian traditional cheese characterized by the squeaking property during consumption. It was assumed that this ratio would not significantly change the squeaking property, which is the most important characteristic of this cheese. A lower fat/casein ratio allows more trapping of fat into the curd after coagulation that causes more effective fat recovery from milk into cheese. In general, standardization is conducted by the addition of skimmed milk powder (SMP) or skimming part of the milk fat. The use of SMP for the standardization of whole milk for cheese manufacturing offers several advantages including high yield [7]. As well as this, some research has been done with the aim of determining the influence of using ultrafiltered milk retentate, milk protein concentrate and phosphocasein on the quality and characteristics of produced cheese [1,8,9,10]. In contrast, milk standardization is not often carried out in traditional small-scale dairy plant cheese production, which could lead to the reduced recovery of milk fat to cheese and so lesser financial profit. Besides the financial benefit, the purpose of milk standardization is to have consistent cheese quality, especially when seasonal variations are considered [11].

Lički škripavac is Croatian soft unripened cheese, which is characterized by the plastic-pliable texture and, therefore, squeaking property during consumption. The squeaking property of this cheese is a consequence of modest drainage of the whey, high temperature (up to 45 °C) of cooking the curd grain and absence of fermentation. Properly vacuum-packed and stored cheese (4–8 °C) retains its freshness and squeaking property during consumption to the end of shelf life, which could be up to one month [12]. The reputation of this cheese is very high throughout Croatia. Lički škripavac cheese is produced in the mountainous area of Lika. Its name, Lički škripavac, is granted with Protected Geographical Indication (PGI). This cheese is produced mainly from raw milk from cows in extensive feeding conditions with the use of a large proportion of forage of a characteristic natural botanical composition of meadows and pastures and a low share of concentrates. Due to that fact, milk composition for Lički škripavac cheese making is characterized by high fat content and a low protein content [13,14]. Therefore, in the production of such cheeses, milk standardization could be an appropriate way to increase the proportion of casein in relation to fat and negate the unfavorable composition of milk for cheesemaking. So, it would be extremely useful for cheesemakers to control the protein/fat ratio in cheese milk [15]. Standardization of milk protein to higher-than-normal levels offers the advantage of increasing plant output without investment of capital expenditure on extra cheese vats [1].

The aim of this study was to determine the effect of the milk standardization, which included the addition of skimmed milk powder to cheese milk and skimming part of the milk fat, on the chemical composition, fat and protein recovery, yield and sensory properties of Lički škripavac cheese.

## 2. Materials and Methods

### 2.1. Production of Lički Škripavac Cheese

In a small-scale dairy plant in the County of Karlovac, 10 batches of Lički škripavac cheese were produced. The standardization of milk was carried out in two ways: by adding skimmed milk powder to cheese milk and by skimming part of the milk fat. The quantity of added skimmed milk powder and separated cream were obtained by calculation, in accordance with the results of chemical analysis of milk (protein and milk fat content). Therefore, milk from each batch was divided into three portions: (1) milk for cheese production without standardization (control group); (2) milk for cheese production with standardized casein-to-fat ratio of 0.7 by the addition of SMP. SMP was purchased from the local market and contained 1.5 g of fat, 50 g carbohydrates, 34 g protein and 1 g of salt in 100 g of product. It was directly added and steadily mixed to completely dissolve in warm milk (35 °C) before renneting; (3) milk for cheese production with standardized casein-to-fat ratio of 0.7:1 by skimming part of the milk fat by centrifugal separation.

Lički škripavac cheese was made from raw milk (a mixture of evening and morning milk) heated up to a temperature of 35 °C. In warm milk, the powder rennet (Caglificio Clerici, Cadorago, Italy; strength 890 IMCU/g) was added (previously dissolved in warm water), stirred for 3–5 min. Once rennet was added into milk, it was left to stand for 40 min, and then the quality of the coagulum was checked. When coagulum was firm enough during test cutting, and when it appeared that the whey was transparent, it was decided that coagulation was completed and coagulum was ready for cutting. The curd was manually cut by a sharp harp into cubes (size 2 × 2 cm), stirred for 5–7 min and heated gradually to 45 °C (1 °C/2 min). At this temperature, the curd was left for another 10 min, then whey was drained, while curd grains were directly salted (2% of added salt), placed in molds and modestly pressed for about 1 h. After pressing, cheeses were kept overnight under refrigerator temperature (4–8 °C). Cold PGI Lički škripavac cheese does not release the whey. The next day cheese was weighted and analyzed. All cheese-making steps were conducted according to the operations described in PGI standard (Croatian Ministry of Agriculture, 2019) that are applied in all Croatian dairies that produce this type of cheese [16].

### 2.2. Physicochemical Analysis of Milk, Cheese and Whey

The physicochemical analysis of milk, cheese and whey were conducted at the Reference Laboratory for Milk and Milk Products of the Department of Dairy Science at the University of Zagreb Faculty of Agriculture. The content of milk fat, protein, lactose, non-fat total solids in milk and whey were measured on the instrument MilkoScan FT 120 (FOSS, Hillerød, Denmark) using infra-red spectrometry [17]. The proportion of casein in milk was determined by the precipitation of casein at pH = 4.6 by direct reference method [18].

Analyses of cheese included determination of fat content according to the Van Gulik method [19], protein content according to Kjeldahl [20], total solids [21], pH value (Mettler Toledo, Seven Multi, according to manufacturer’s instructions) and salt content according to the potentiometric titration method [22].

### 2.3. Cheese Yield, Fat and Protein Recovery Calculation

Actual cheese yield for each batch was calculated by dividing the weight of cheese after production by the weight of cheese milk. Moisture- and salt-adjusted cheese yield (ACY) was calculated using an Equation (1) with desired cheese moisture of 49.1% and desired cheese salt content of 0.87% [23].

(1) $$\mathrm{AJY}\;=\;\frac{\mathrm{Actual}\;\mathrm{Yield}\;\left[100-\left(\mathrm{Actual}\;\%\;\mathrm{moisture}+\%\;\mathrm{salt}\right)\right]\;}{100-\;\left[\mathrm{Desired}\;\%\;\mathrm{moisture}+\%\;\mathrm{salt}\right]}$$

Percentage of fat recovery (2) and casein recovery (3) in each cheese was calculated according to [24]:

(2) $$\mathrm{RF}\;=\;\frac{\left(\%\;\mathrm{fat}\;\mathrm{in}\;\mathrm{cheese}\;\times \;\mathrm{cheese}\;\mathrm{wt}\right)}{\left(\%\;\mathrm{fat}\;\mathrm{in}\;\mathrm{milk}\;\times \;\mathrm{milk}\;\mathrm{wt}\right)}$$

(3) $$\mathrm{RC}\;=\;\frac{\left(\%\;\mathrm{casein}\;\mathrm{in}\;\mathrm{cheese}\;\times \;\mathrm{cheese}\;\mathrm{wt}\right)}{\left(\%\;\mathrm{casein}\;\mathrm{in}\;\mathrm{milk}\;\times \;\mathrm{milk}\;\mathrm{wt}\right)}$$

where RF is the fat recovered in cheese, and RC is the casein recovered in cheese.

### 2.4. Sensory Evaluation of Lički Škripavac Cheese

To determine the influence of standardization of milk on the sensory properties of Lički škripavac cheese, a blind test was used, which included cheese tasting by a group of trained participants (*n* = 17) who are familiar with the properties of Lički škripavac cheese, whereby they filled out a questionnaire. The participants had to taste three different groups of Lički škripavac cheese samples: cheese without standardization (control group), cheese standardized by addition of SMP and the cheese standardized by skimming part of the milk fat coded with three different letters. Their task was to answer the question: do they recognize the difference in sensory characteristics between cheeses, and if yes, according to which attributes (color, odor, taste), as well as to evaluate their overall liking of the three cheeses on a scale of 1 to 5 (1 = unacceptable and 5 = very acceptable).

### 2.5. Statistical Analysis

Analysis of variance (ANOVA) was used to test the influence of milk standardization on chemical composition, fat and protein recovery, yield and sensory properties of Lički škripavac cheese. ANOVA procedure was carried out in SPSS [25].

## 3. Results and Discussion

### 3.1. Physicochemical Composition of Milk, Whey and Cheese

The physicochemical composition of milk before standardization is presented in Table 1.

Considering the fact that fat and protein recovery, as well as yield efficiency, decrease as a result of high somatic cell counts (SCC; >400,000/mL; [23]), all batches were produced from milk with SCC below 400,000/mL. Moreover, beside lactose content, all cheese milk samples within the ranges reported by others [11,26] but below of optimal casein-to-fat ratio of 0.7 proposed by Scott [6] are shown in Table 1. Taking into consideration that SCC was below 400,000/mL, the lower content of lactose was probably due to storage time of milk before processing. However, pH values of milk samples were above 6.6, which confirmed that all milk samples were suitable for making cheese [11].

When the protein (casein)-to-fat ratio is below optimal, the losses of fat by whey increase [1] due to the lower content of casein in relation to the fat that causes less efficient fat trapping during the coagulation process [6]. Results obtained by this research showed that losses of fat by whey were significantly (*p* < 0.05) lower after Lički škripavac cheese making from standardized skimmed milk (Table 2). These results are in accordance with the results of Addis et al. [15] who found there to be lower fat content in the whey after production of Protected Denomination of Origin (PDO) Pecorino Romano cheese from standardized cheese milk (protein/fat = 0.9) by addition skimmed sheep milk into full fat sheep milk. On the contrary, standardization of cheese milk by addition of SMP caused higher losses of protein (*p* < 0.05) and total solids (*p* < 0.0001). Whey obtained after Lički škripavac cheese making from standardized cheese milk by addition of SMP contained a higher content of lactose (*p* < 0.0001), as shown in Table 2.

Table 3 shows that the composition of Lički škripavac cheese made by three different treatments was within the ranges of literature values for Lički škripavac cheese [13,14]. However, both methods of cheese milk standardization caused a significant (*p* < 0.01) decrease in milk fat content and fat in dry matter in Lički škripavac cheese, which is in accordance with Addis et al. [15] who found that standardization of milk protein-to-fat ratio by addition of an amount of skimmed sheep milk lowers fat content and fat in dry matter in PDO Pecorino Romano cheese. Moreover, Francolino et al. [27] reported less fat content in Mozzarella cheese produced from standardized cheese milk by addition of protein concentrate. In contrast, standardization of cheese milk caused a significant (*p* < 0.01) increase in the protein content in cheese milk. This is in line with Pappas et al. [28] who found that Feta cheese produced from standardized ewe’s milk with 0.8 casein/fat had a higher protein content than the cheeses produced from the lower casein/fat ratio (<0.72). Similar results were obtained by Addis et al. [15] who determined higher protein content in PDO Pecorino Romano cheese produced from standardized protein-to-fat sheep milk (0.9) in comparison to the natural (0.76) protein-to-fat ratio of sheep milk. These differences could be attributed to the differences in relative proportion of casein-to-fat in milk. On the contrary, Shakeel-Ur-Rahman et al. [8] reported that mean compositions of 40 to 50% reduced fat Cheddar cheese produced from whole milk, which is standardized with dry milk protein concentrate or skimmed milk, were not different.

Total solids of Lički škripavac cheese did not significantly change (*p* = 0.11) as a consequence of increasing the content of milk protein in cheese milk by addition of skimmed milk powder (treatment 2), which is in contrast to the finding of Guinee et al. [1], who found that the level of cheese moisture decreased in increasing milk protein level, and Brito et al. [29], who reported lower moisture contents of semi-hard Maribo cheese made with addition of milk powder. Both types of standardization of casein/fat ratio to 0.7 reduced variability of fat in dry matter content in cheeses by decreasing it for almost 4% (*p* < 0.01) in comparison to the control cheese. Moisture in non-fat substance (MNFS) significantly (*p* < 0.05) decreased as a consequence of increased casein/fat ratio, which is in accordance with Addis et al. [15], but contrary to the results of Pappas et al. [28], who did not find an effect of different casein/fat ratio on MNSF of Feta cheese produced from sheep milk. Fat mechanically blocks the casein–casein interactions that are likely to be partly responsible for less intensive syneresis of cheese [28]. The salt-in-moisture contents (S/M) were not significantly changed as a consequence of casein/fat standardization. Pappas et al. [28] determined decreasing of S/M for Feta cheese, as well as Addis et al. [15] for PDO Pecorino Romano cheese, which could be connected to the fact that those cheeses were subjected to the ripening process. The pH of cheese was affected by casein/fat ratio only when Lički škripavac cheese was standardized by skimming part of the milk fat, which is in contrast with the findings of Pappas et al. [28]. This can be explained by partial removal of milk fat from cheese milk and low salt in moisture content. Thomas and Pearce [30] reported that acid development by starter cultures and non-starter lactic acid bacteria were not affected by a low level (<4%) of salt in moisture. According to this, action of non-starter lactic acid bacteria in this research was not limited by salt in moisture content in cheese. It is also important to emphasize that fat globules had an obstructive effect, which caused a decrease in whey drainage and, by that, also led to reduction in the extent of protein-to-protein interaction within the paracasein network [31]. Since cheeses were produced from unpasteurized skimmed milk, it is assumed that they had a more compact protein structure with low porosity and permeability, which led to significantly (*p* < 0.05) lower MNFS (Table 3). Considering these facts, cheeses with lower MNFS and non-limited activity of starter and non-starter lactic acid bacteria had lower pH as a consequence of the concentration effect of lactic acid.

### 3.2. Fat and Protein Recovery to Lički Škripavac Cheese and Cheese Yield

The fat and protein recovery and actual and adjusted cheese yield to 49.1% of moisture content and 0.87% of salt content are presented in Table 4. Fat recoveries in Lički škripavac cheeses were noticeably lower in comparison to the fat recoveries (91–93%) of reduced-fat Cheddar cheese [8]. In spite of the fact that optimization of the casein/fat ratio did not cause a significant increase in fat to Lički škripavac cheese, such as was found previously [1,15], an increase in fat recovery to the cheese was noticeable [0.51% (for addition SMP) and 0.34% (for skimming part of the milk fat)]. Higher efficiency for 2% of fat recovery as a consequence of addition of milk protein concentrate was found by Shakeel-Ur-Rehman et al. [8]. Pappas et al. [28] reported that the amount of cheese per kg of milk fat decreased as the percentage of fat in milk increased. Protein recovery from milk to cheese significantly (*p* < 0.01) increased; 2.1% for addition of SMP and 1.88% for skimming part of the milk fat, which is in accordance with the finding of Guinee et al. [1] who found that increasing in milk protein level from 3.3 to 4.0% (wt./wt.), increased the percentage of milk protein recovered to cheese.

The actual yields obtained in this study are lower in comparison to similar semi soft varieties of cheese. Thus, Hayaloglu et al. [32] reported that the average yield of Turkish Beyaz Peynir cheese made from cow’s milk is 15–16 kg/100 kg. The addition of SMP to cheese milk to optimize the casein/fat ratio significantly (*p* < 0.01) increased actual and adjusted cheese yield (Table 4). This is in accordance with Shakeel-Ur-Rehman et al. [8] who reported that addition of milk protein concentrates doubled cheese yield due to the high recovery of milk solids in cheese. Moreover, Francolino et al. [27] reported that yield increased due to the higher recovery of the milk total solids and protein in Mozzarella cheese produced by addition of milk protein concentrate to cheese milk. However, the removal of milk fat generally results in decreased yields [8,15,28], which is confirmed by this investigation too (Table 4; *p* < 0.01). Moreover, our finding is in accordance with the findings of Guinee et al. [1], who reported the increase in the actual and moisture-adjusted yield with increase in milk protein level.

### 3.3. Sensory Evaluation

**Figure 1:**
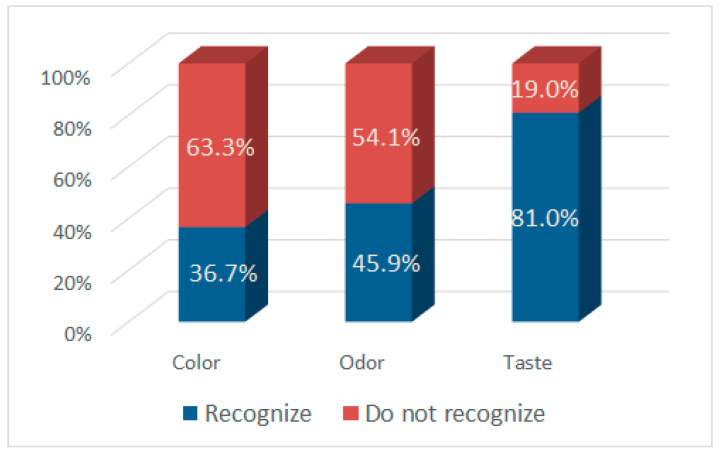
Consumer’s recognition of difference in color, odor and taste between cheeses.

**Figure 2:**
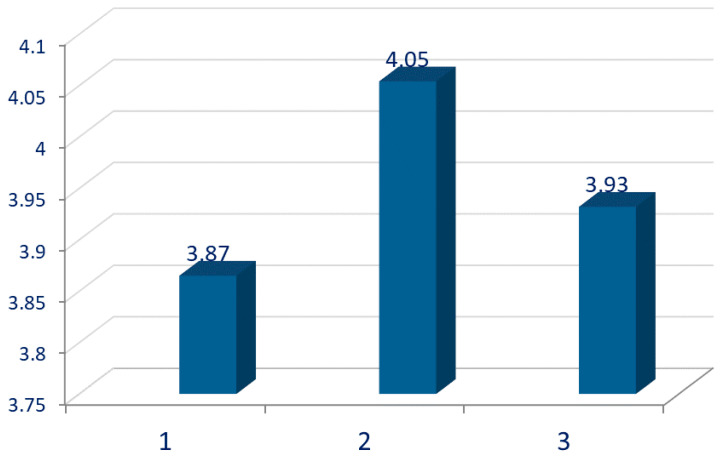
Consumer’s average sensory score of evaluated cheeses (*p* = 0.10) using a scale of 1 to 5 (1 = unacceptable and 5 = very acceptable). 1—without milk standardization; 2—standardization casein/fat = 0.7 by addition of skimmed milk powder; 3—standardization casein/fat = 0.7 by skimming part of the milk fat.

The influence of standardization of milk on the sensory properties of Lički škripavac cheese was done by using a blind test as presented in Figure 1 and Figure 2. More than 80% of evaluators recognized differences in the taste between cheeses, while the percentage of recognition of difference for color and odor is much lower. The addition of SMP led to a noticeably higher (*p* = 0.10) sensory score, which is in contrast to Anderson et al. [33] who reported that the addition of condensed skimmed milk leads to an atypical flavor in cheese. Addis et al. [15] reported that skimming of cheese milk does not have a direct effect on flavor development of PDO Pecorino Romano cheese. Moreover, Shakeel-Ur-Rehman et al. [8] also reported that the score for milk fat flavor was lower in the reduced-fat Cheddar cheese produced from standardized milk (casein/fat = ~1.8) obtained by mixing whole milk and commercial milk protein concentrate. On the other hand, within the range of protein percentages studied (0.9, 1.8, 2.8, 3.7 and 4.6%), no changes in flavor and aroma descriptors were detected [34]. Pappas et al. [28] reported that the sensory properties of Feta cheese produced from sheep milk with casein/fat ratio of 0.62 has better sensory quality in comparison to cheese produced from the sheep milk with a 0.8 casein/fat ratio. These differences could be due to different types of analyzed cheeses. The addition of SMP into cheese milk for Lički škripavac cheese making probably emphasized some sensory attributes of this cheese, such as full milky flavor, because this is an unripened type of cheese and its flavor directly depends on the flavor of milk used for its production [14].

## 4. Conclusions

This study demonstrated the feasibility of standardizing casein/fat ratio of cheese milk by the addition of skimmed milk powder or by skimming part of the milk fat. By both types of standardization, milk fat content in Lički škripavac cheese decreased, while the content of protein increased, which resulted in higher actual and adjusted yield, as well as higher consumer’s sensory score. The addition of skimmed milk powder in the production of soft unripened cheeses such as Lički škripavac cheese probably contributes to its full milky flavor and better squeaking property. These attributes are the most important sensory attributes of Lički škripavac cheese. However, further research of textural properties of Lički škripavac cheese produced from reformulated milk are needed.

## Figures and Tables

**Table 1:** Physicochemical composition of cheese milk before standardization (*n* = 10).

Milk Fat (g/100 g)	Protein (g/100 g)	Casein/Fat	Casein (g/100 g)	Lactose (g/100 g)	Total Solids (g/100 g)	Solids Non-Fat (g/100 g)	SCC (10^3^)	pH
3.98	3.15	0.64	2.54	4.31	12.37	8.38	145	6.71
3.92	3.16	0.63	2.48	4.34	12.35	8.42	141	6.61
4.11	3.15	0.59	2.43	4.36	12.58	8.44	87	6.71
4.07	3.24	0.62	2.53	4.27	12.55	8.43	149	6.70
4.07	3.31	0.62	2.52	4.26	12.61	8.49	130	6.62
4.07	3.33	0.63	2.54	4.26	12.62	8.51	122	6.67
4.29	3.50	0.64	2.75	4.33	13.08	8.76	159	6.60
4.67	3.48	0.58	2.70	4.31	13.41	8.71	205	6.61
5.21	3.69	0.55	2.85	4.04	13.89	8.64	332	6.66
4.67	3.49	0.57	2.66	4.09	13.21	8.49	273	6.71

SCC = somatic cell counts.

**Table 2:** The composition of whey obtained after Lički škripavac cheese production regarding different type of cheese milk standardization (least square means ± standard errors).

Treatment	Total Solids (g/100 g)	Milk Fat (g/100 g)	Protein (g/100 g)	Lactose (g/100 g)
1	7.73 ^A^ ± 0.11	1.34 ^a^ ± 0.08	1.02 ^a^ ± 0.02	4.42 ^A^ ± 0.05
2	8.58 ^B^ ± 0.11	1.43 ^a^ ± 0.08	1.12 ^b^ ± 0.02	5.09 ^B^ ± 0.05
3	7.58 ^A^ ± 0.11	1.16 ^b^ ± 0.08	1.03 ^a^ ± 0.02	4.43 ^A^ ± 0.05

^a, b^ Means within the same column marked with the different letter differ significantly (*p* < 0.05). ^A, B^ Means within the same column marked with the different letter differ significantly (*p* < 0.0001). 1—without milk standardization; 2—standardization casein/fat = 0.7 by addition of skimmed milk powder; 3—standardization casein/fat = 0.7 by skimming part of the milk fat.

**Table 3:** Effect of milk standardization on physicochemical characteristics of Lički škripavac cheese (least square means ± standard errors).

Parameter	Treatments			*p* Value
	1	2	3	
Milk fat (g/100 g)	26.60 ^a^ ± 0.28	24.22 ^b^ ± 0.27	25.00 ^b^ ± 0.27	<0.01
Protein (g/100 g)	19.29 ^a^ ± 0.22	20.49 ^b^ ± 0.22	21.34 ^c^ ± 0.21	<0.01
Total solids (g/100 g)	50.95 ± 0.39	50.28 ± 0.39	51.46 ± 0.38	*p* = 0.11
Fat in dry matter (%)	52.21 ^a^ ± 0.55	48.22 ^b^ ± 0.54	48.62 ^b^ ± 0.55	<0.01
MNFS (%)	66.82 ^a^ ± 0.49	65.64 ^b^ ± 0.49	64.72 ^c^ ± 0.49	<0.05
NaCl (g/100 g)	0.76 ± 0.06	0.93 ± 0.06	0.90 ± 0.06	*p* = 0.16
S/M (%)	1.56 ± 0.13	1.87 ± 0.12	1.87 ± 0.13	*p* = 0.18
pH	6.48 ^a^ ± 0.06	6.46 ^a^ ± 0.06	6.27 ^b^ ± 0.06	*p* < 0.05

^a, b, c^ Means within the same row marked with the different letter differ significantly (*p* < 0.05). MNFS = moisture in non-fat substance. S/M = salt-in-moisture content. 1—without milk standardization; 2—standardization casein/fat = 0.7 by addition of skimmed milk powder; 3—standardization casein/fat = 0.7 by skimming part of the milk fat.

**Table 4:** Effect of milk standardization on fat and protein recovery to Lički škripavac cheese and actual and adjusted cheese yield (least square means ± standard errors).

Parameter	Treatments			*p* Value
	1	2	3	
Fat recovery (% of total milk fat)	80.14 ± 1.50	80.65 ± 1.50	80.48 ± 1.50	*p* = 0.97
Protein recovery (% of total milk protein)	74.41 ^a^ ± 0.85	76.51 ^ab^ ± 0.85	76.72 ^b^ ± 0.85	<0.05
Actual yield (kg cheese/kg cheese milk × 100)	12.91 ^a^ ± 0.22	14.22 ^b^ ± 0.22	11.95 ^c^ ± 0.21	<0.01
Adjusted yield * (%)	12.95 ^a^ ± 0.25	14.04 ^b^ ± 0.25	12.07 ^c^ ± 0.24	<0.01

* to 49.1% moisture content and 0.87% salt content. ^a, b, c^ Means within the same row marked with the different letter differ significantly (*p* < 0.05). 1—without milk standardization; 2—standardization casein/fat = 0.7 by addition of skimmed milk powder; 3—standardization casein/fat = 0.7 by skimming part of the milk fat

## Data Availability

Not applicable.
